# Editorial: Exploring the interface of mucosal vaccines and immune system modulation

**DOI:** 10.3389/fcimb.2026.1875392

**Published:** 2026-06-16

**Authors:** Jinyi Tang, Arka Sen Chaudhuri, Bibo Zhu

**Affiliations:** 1 Beirne B. Carter Center for Immunology Research, University of Virginia, Charlottesville, VA, United States; 2 Division of Infectious Disease and International Health, Department of Medicine, University of Virginia, Charlottesville, VA, United States; 3 National Key Laboratory of Agricultural Microbiology, College of Veterinary Medicine, Huazhong Agricultural University, Wuhan, Hubei, China

**Keywords:** adjuvant, *ex vivo* mucosal cultures, mucosal IgA, mucosal T cells, mucosal vaccines

Mucosal surfaces are the primary entry points for most infections. Yet, historically, the bulk of our prophylactic strategies have relied on systemic immunization. While often effective at preventing severe systemic disease, intramuscular or subcutaneous vaccines frequently fall short in establishing the localized, tissue-resident immunity necessary to block initial infection and transmission at the mucosal barrier. Achieving true sterilizing immunity requires engaging the mucosal immune system directly, specifically through the induction of secretory IgA (sIgA) and the establishment of localized T and B cell memory. The core challenge in mucosal vaccinology is not simply delivering the antigen but overcoming the inherent tolerogenic baseline of the mucosal microenvironment safely and effectively. This Research Topic, “Exploring the Interface of Mucosal Vaccines and Immune System Modulation,” brings together recent work focused on how targeted immunological interventions can bypass these barriers to elicit durable protection at our most vulnerable physiological borders. This Research Topic highlights the identification and application of novel mucosal adjuvants. A central challenge remains: how to effectively engage local antigen-presenting cells without triggering harmful inflammation.

Puente-Marín et al. demonstrated the efficacy of the STING agonist cyclic di-adenosine monophosphate (c-di-AMP) as a potent mucosal adjuvant in a large mammalian model. By sublingually co-administering c-di-AMP with the chimeric E2-CD154 protein against Classical Swine Fever Virus (CSFV), they induced robust systemic IgG and IgA responses, effectively restricting viral replication and completely preventing the recovery of infectious virus from tonsillar tissue. Importantly, this mucosal approach achieved protection comparable to established intramuscular vaccines while keeping innate inflammatory markers, such as serum IFN-α, minimal post-challenge.

Beyond adjuvant selection, identifying antigens capable of driving specific T helper cell polarization is key to shaping the architecture of the downstream adaptive response. Xie et al. evaluated several immunodominant proteins of Mycoplasma hyopneumoniae to determine their capacity to elicit comprehensive immunity. Their findings highlight that the recombinant protein Mhp274 acts as a viable subunit vaccine candidate by successfully driving a balanced Th1/Th2/Th17 immune response, evidenced by the secretion of IFN-γ, IL-4, and IL-17, and uniquely stimulating the significant production of local sIgA in bronchoalveolar lavage fluid.

This Research Topic also underscores the interconnected nature of the common mucosal immune system, wherein immunization at one inductive site can confer effector protection at a distant mucosal tissue. Ma et al. explored this cross-talk by utilizing an attenuated Chlamydia muridarum mutant (CM-pGP3S) as a live rectal vaccine to defend against genital tract infections. Rectal inoculation elicited robust transmucosal immunity, significantly reducing the duration of vaginal shedding and completely preventing severe upper genital tract pathology (hydrosalpinx) following wild-type challenge, notably without inducing colitis or disrupting the gastrointestinal microbiota.

Finally, a persistent bottleneck in translating these mucosal strategies to the clinic is the poor predictive value of traditional animal models, which often fail to mirror human mucosal architecture and innate signaling pathways. Chilakamarri et al. provided a comprehensive review of the rapid advancements in human ex vivo mucosal cultures. From organotypic air-liquid interface (ALI) cultures to complex lymphoid organoids and microfluidics-based organ-on-a-chip systems, these emerging platforms offer a more biologically accurate environment to evaluate antigen presentation, adaptive immune priming, and host-microbiome interactions, serving as essential tools for the next-generation of vaccine development.

These articles argue that moving beyond systemic immunization requires a more deliberate engagement with the mucosal niche. As we refine our understanding of how delivery systems and adjuvants interact with local immune memory, and as human ex vivo models become more sophisticated, we come closer to developing vaccines that neutralize infections exactly where they begin.

